# Towards personalized medicine with iPS cell technology: a case report of advanced systemic mastocytosis with associated eosinophilia

**DOI:** 10.1007/s00277-022-04975-9

**Published:** 2022-09-20

**Authors:** Salim Atakhanov, Deborah Christen, Benjamin Rolles, Herdit M. Schüler, Jens Panse, Nicolas Chatain, Steffen Koschmieder, Tim H. Brümmendorf, Marcelo A. S. Toledo, Martin Zenke

**Affiliations:** 1 Institute for Biomedical Engineering, Department of Cell Biology, RWTH Aachen University Medical School, Aachen, Germany; 2 Helmholtz Institute for Biomedical Engineering, RWTH Aachen University, Aachen, Germany; 3 Institute for Cell and Tumor Biology, RWTH Aachen University Medical School, Aachen, Germany; 4 Department of Hematology, Oncology, Hemostaseology and Stem Cell Transplantation, Faculty of Medicine, RWTH Aachen University, Aachen, Germany; 5 Center for Integrated Oncology, Aachen Bonn Cologne Düsseldorf (CIO ABCD), Aachen, Germany; 6 Division of Hematology, Department of Medicine, Brigham and Women’s Hospital, Harvard Medical School, Boston, MA USA; 7 Institute for Human Genetics, RWTH Aachen University Hospital, Aachen, Germany

Dear Editor,

Advanced systemic mastocytosis (advSM) often occurs with concurrent eosinophilia [1, 2]. Here, we report a case of advSM with associated eosinophilia and the generation of patient-specific induced pluripotent stem cells (iPS cells) as an attractive, personalized approach for compound identification to overcome unresponsiveness to anti-neoplastic treatment.

**Fig. 1:**
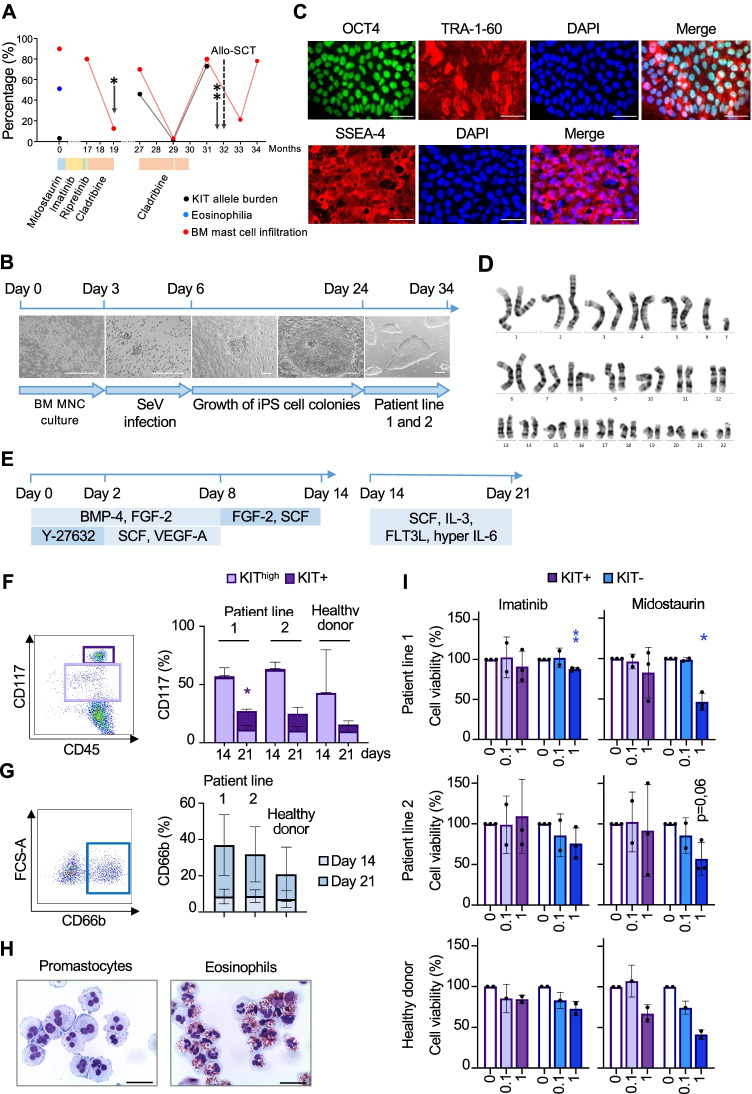
Advanced systemic mastocytosis (advSM) patient iPS cells and their differentiation for compound screening. **A** Overview of the clinical data of the advSM patient. *X* axis represents months after initial diagnosis (February 2018). The graph shows KIT allele burden (black), the frequency of eosinophils (blue) and mast cells (red) throughout the patient’s disease history. BM biopsy was performed (one asterisk) and BM mononuclear cells were used for iPS cell generation, differentiation and compound testing (two asterisks) as indicated. **B** Schematic overview of iPS cell generation. Bone marrow mononuclear cells (BM MNC) were cultured for 3 days with SCF, Flt3 ligand (Flt3L), TPO, and hyper-IL-6. Cells were then subjected to infection with Sendai virus (SeV) vectors with reprogramming factors and the formation of cell clusters was observed at day 6, indicating successful reprogramming. Cells were seeded on mouse embryonic fibroblasts (MEF) feeder and selected iPS cell colonies were manually picked at day 24 and further expanded for genotyping and establishment of stable iPS cell lines. Scale bars: 200 µm. **C** Representative images of immunofluorescence staining for pluripotency markers OCT4, TRA-1–60, and SSEA-4. DAPI was used to stain nuclei. Merge represents overlay of pluripotency markers and DAPI. Scale bars: 50 µm. **D** Representative image of cytogenetic analysis for patient-derived iPS cell lines. Karyotyping of iPS cell lines shows normal male karyotype (46,XY) with no numeric or structural aberrations. **E** Schematic representation of spin embryonic body (spin EB) protocol for hematopoietic differentiation. iPS cell lines were differentiated towards hematopoietic progenitors using a protocol modified from Liu et al. [5]. Cell culture medium was sequentially supplemented with cytokines as shown. On day 14, cells were harvested for further analysis and expanded until day 21 in suspension culture with the indicated cytokine cocktail. On day 21, cells were harvested and analyzed. **F** Analysis of KIT population throughout the differentiation. Representative flow cytometry plot (left) of CD45^+^CD117^+^ and CD45^+^KIT^high^ (promastocytes) populations on day 21. The frequency of both populations on days 14 and 21 for patient and healthy donor [6] iPS cell-derived cells are illustrated in graphs (right). Data demonstrate the mean ± SD of the percentage of populations. Statistical significance using Welch’s *t* test was compared to the healthy donor iPS cell-derived cells. *:*p* ≤ 0.05, *n* = 7. **G** Analysis of granulocytic population throughout the differentiation. Representative flow cytometry plot (left) of CD45^+^CD117^−^CD66b^+^ populations on day 21. The frequency of populations on days 14 and 21 for patient and healthy donor iPS cell-derived cells are illustrated in graphs (right). Data demonstrate the mean ± SD of the percentage of populations (*n* = 8). **H** Representative images of myeloid cells upon differentiation. Cytospin preparations of CD45^+^CD117^+^ (left) and CD45^+^CD117^−^ (right) on day 21 of directed differentiation show promastocytes and eosinophils, respectively. Acidic blue staining was performed to stain KIT^+^ cells and Diff-Quik staining was performed to stain KIT^−^ population. Scale bars: 30 µm. **I** Compound testing on iPS cell-derived hematopoietic progenitors. Average drug response ± SD for KIT^+^ (purple) and KIT.^−^ (blue) of patient and healthy donor iPS cell-derived cells. Each dot represents one independent experiment. 1 µM imatinib and midostaurin and untreated controls (0 µM): patient lines 1 and 2, *n* = 3; healthy donor *n* = 2; 0.1 µM imatinib and midostaurin: patient lines 1 and 2, *n* = 2; healthy donor, *n* = 1; Concentrations are indicated in µM. Statistical significance using Welch’s t-test was compared to control (0 µM). *:*p* ≤ 0.05, **: *p* ≤ 0.01

A 45-year-old male patient was diagnosed with mast cell leukemia (MCL) in February 2018 (Fig. 1A; month 0) and referred to our center in April 2019. Bone marrow (BM) smears at presentation showed 80% mast cell infiltration. Blood counts revealed leukocytosis (white blood cells 19,000/µl) with eosinophilia (51.4%) and anemia (Hb = 8.1 g/dl) but normal platelet counts. Next generation sequencing analysis [3] detected *KITS476I* (c.1427G > T, 3.1%) and *KRASG12V* (c.35G > T, 7.3%) mutations. Fluorescent in situ hybridization (FISH) analysis revealed a PDGFRß rearrangement (5q32) in 1% of the interphases, while cytogenetics showed a normal male karyotype (46,XY). Successive treatments with midostaurin, imatinib, and ripretinib from February 2018 to July 2019 (month 0–17) resulted only in brief periods of clinical improvement. From July to August 2019 (month 17–19), he received 2 cycles of cladribine, resulting in partial remission according to IWG-MRT ECNM consensus response criteria [4]. At this time, he was considered for allogeneic stem cell transplantation (allo-SCT) but avoided follow-up visits during the first SARS-CoV-2 pandemic wave.

In May 2020 (month 27), he presented with progressing MCL. Three cycles of cladribine failed to achieve any relevant clinical improvement. Lacking alternative options, allo-SCT from a haploidentical-related donor after conditioning with fludarabine (50 mg/m^2^), thiotepa (5 mg/m^2^), and busulfan (0.66 mg/m^2^) was performed without acute complications. On day 50, post-allo-SCT BM biopsy showed 78% mast cells, and the patient died within a few days due to multiorgan dysfunction.

In parallel to conventional therapy with the aim to identify compounds that would potentially be effective for the patient, we generated iPS cells from the patient´s BM mononuclear cells (Fig. 1A, B). Two iPS cell lines were further studied and displayed the characteristic pluripotent phenotype (Fig. 1C). Karyotype analysis showed no chromosomal abnormalities (Fig. 1D). The KIT S476I mutation was not detected in the iPS cell clones due to the low allele burden. Importantly, iPS cell-derived hematopoietic cells recapitulated the mast cell (CD45^+^KIT^high^) and granulocytic (CD45^+^CD66b^+^) bias of the disease (Fig. 1E–H). This allowed us to proceed with the screening of therapeutic compounds within only 2.5 months after iPS cell generation (Fig. 1I). Imatinib (1 µM) and midostaurin (1 µM) were essentially ineffective on CD45^+^KIT^+/high^ hematopoietic cells, in agreement with the patient’s clinical data. Unfortunately, the patient died before further compound screenings were finalized.

In summary, within 2.5 months, iPS cell-derived patient-specific drug response data were obtained. This highlights iPS cells as a powerful tool for personalized medicine in oncological hematology when anti-malignant treatment options are exhausted. In addition, we expect a library of patient-specific iPS cell lines with SM mutations and automation of iPS cell production to further accelerate the identification of patient tailored therapies [7–9].
